# Success stories of natural product-derived compounds from plants as multidrug resistance modulators in microorganisms

**DOI:** 10.1039/d3ra00184a

**Published:** 2023-03-08

**Authors:** Xiaohan Zhai, Guoyu Wu, Xufeng Tao, Shilei Yang, Linlin Lv, Yanna Zhu, Deshi Dong, Hong Xiang

**Affiliations:** a Department of Pharmacy, First Affiliated Hospital of Dalian Medical University Dalian China dongdeshi@dmu.edu.cn xianghong@dmu.edu.cn; b Laboratory of Integrative Medicine, First Affiliated Hospital of Dalian Medical University Dalian China

## Abstract

Microorganisms evolve resistance to antibiotics as a function of evolution. Antibiotics have accelerated bacterial resistance through mutations and acquired resistance through a combination of factors. In some cases, multiple antibiotic-resistant determinants are encoded in these genes, immediately making the recipient organism a “superbug”. Current antimicrobials are no longer effective against infections caused by pathogens that have developed antimicrobial resistance (AMR), and the problem has become a crisis. Microorganisms that acquire resistance to chemotherapy (multidrug resistance) are a major obstacle for successful treatments. Pharmaceutical industries should be highly interested in natural product-derived compounds, as they offer new sources of chemical entities for the development of new drugs. Phytochemical research and recent experimental advances are discussed in this review in relation to the antimicrobial efficacy of selected natural product-derived compounds as well as details of synergistic mechanisms and structures. The present review recognizesand amplifies the importance of compounds with natural origins, which can be used to create safer and more effective antimicrobial drugs by combating microorganisms that are resistant to multiple types of drugs.

## Abstract 2

Microorganisms evolve resistance to antibiotics as a function of evolution. Antibiotics have accelerated bacterial resistance through mutations and acquired resistance through a combination of factors.

## Graphical abstract

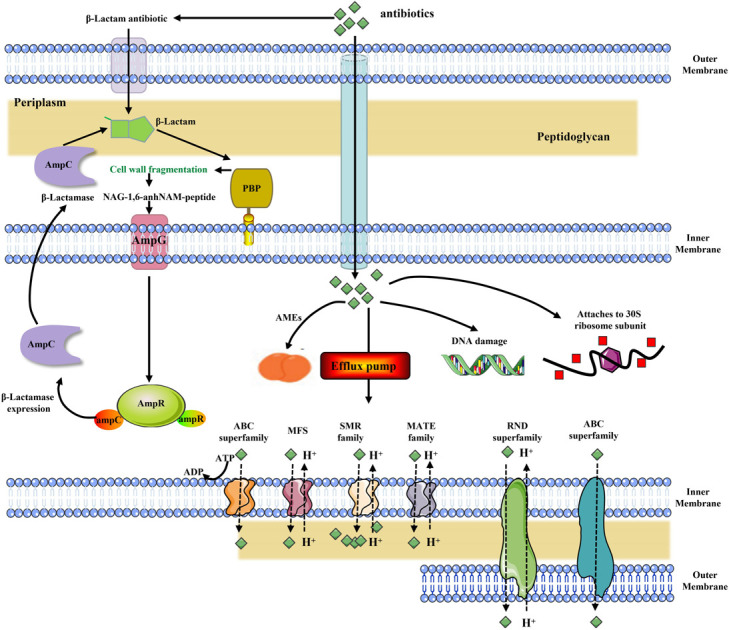

## 1. Introduction

**Fig. 1:**
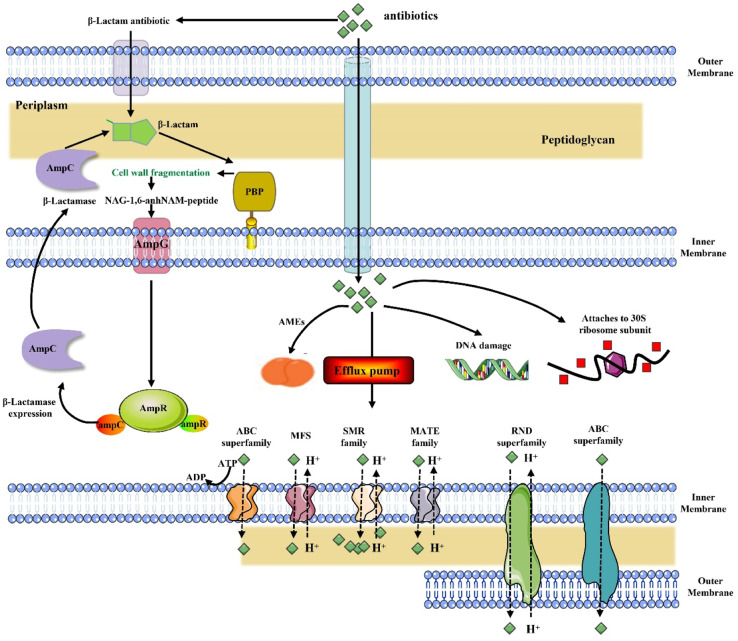
Mechanism of development of multidrug resistance. AMEs: modifying aminoglycoside modifying enzymes; ABC: ATP-binding cassette; MFS: major facilitator superfamily; MATE: multidrug and toxin extrusion; SMR: small multidrug resistance; RND: resistance-nodulation-cell division; PBP: penicillin-binding protein.

Multidrug-resistant microorganisms (MDRMOs) are progressively being regarded as a major global health challenge.^1,2^ As a result of conventional antibiotic pressure, bacterial efflux pumps were increased, reducing drug concentrations; enzymes were induced to modify and inactivate antibiotic compounds; or the drug target site was elevated, reducing antibiotic potency (Fig. 1).^3,4^

Infections by pathogenic bacteria, especially those that are multidrug resistant (MDR), are leading to a growing global health crisis. As a result, MDR bacteria can spread rapidly in hospitals and in the community, posing a staggering burden on health care systems both economically and epidemiologically.^5^ In addition, the inability of current clinical technologies to accurately diagnose infections in a timely manner further aggravates the resistance situation.^6^ By 2050, the number of antimicrobial resistance-related deaths could amount to 10 million, which is far more than the number of cancer deaths.^2^ As we approach the postantibiotic era, normal infections and minor injuries will no longer be treatable. However, in contrast to the rapidly spreading threat of antibiotic resistance, recent decades have seen a dramatic decline in new antibiotic development, with many pharmaceutical companies abandoning antibiotic discovery programs. Due to the subsiding development of antibiotics, alternative therapeutics are needed to treat MDR bacteria, and antimicrobial stewardship necessitates urgent action to stop the evolution of resistance and prevent the emergence of new resistance.

Drug development is aided by natural compounds, especially in regard to the discovery of antimicrobial drugs.^7–9^ Microorganisms have become increasingly resistant and the use of antibiotics is causing numerous side effects, making it essential to develop new and improved antibacterial agents using plant-based derivatives.^10,11^ The purpose of this review is to discuss compounds of natural products and derivatives derived from plants that have been investigated as modulators of MDR by inhibiting or otherwise stopping their activity.

## 2. Molecular antibiotic resistance mechanisms

By overusing antibiotics, microbes have evolved resistance to many conventional antimicrobials. There are several mechanisms that can lead to drug resistance in microbes, including inactivation or modification of antimicrobial drugs, alteration of drug target sites and expulsion of antimicrobial drugs across the cell membrane reduces drug accumulation. The combination of all of these factors contributes to the development of resistance to current antimicrobial therapy.

### 2.1. Alterers: inactivated antimicrobial drugs

#### 2.1.1 β-Lactamases

The most widely used antimicrobial agents are β-lactams. Additionally, Gram-negative bacteria produce β-lactamases to resist β-lactams. To overcome β-lactamase-mediated resistance, a valuable alternative is to develop new β-lactams that are completely stable to the prevalent β-lactamases; however, this goal is likely impossible.^12,13^ To protect β-lactam antibiotics from the activity of β-lactamases, combinations of β-lactams with inhibitors of β-lactamases have been used. Through this strategy, three very successful combination drugs have been produced, namely, ampicillin-sulbactam, amoxicillin-clavulanate, and piperacillin-tazobactam. These drugs are still among the most commonly prescribed antibiotics in clinical settings after two decades of use.^14,15^ There are several advantages to using combinations of β-lactam and β-lactamase inhibitors compared to expanded-spectrum cephalosporins, and these drugs are less likely to result in resistance.

Current therapeutic β-lactamase inhibitors are only effective against class A β-lactamases and do not protect against other β-lactamases. A further challenge for β-lactams and β-lactamase inhibitors is that some strains produce multiple enzymes. Despite reports that new β-lactamase inhibitors are appearing increasingly frequently in the medical literature, some of these inhibitors demonstrate good inhibition properties, as well as inhibiting a variety of β-lactamases simultaneously; other than AVE1330 and NXL104, none of these inhibitors have even reached phase I trials.^16,17^ Molecular modeling and resolution of new lactamase structures may help to overcome one of the most serious threats to human health.

#### 2.1.2 Aminoglycoside modifying enzymes (AMEs)

For decades, aminoglycosides have been an integral part of the arsenal of antibiotics used to treat life-threatening infections. Sadly, their effectiveness has been diminished by surges in resistance and dissemination. Occasionally, their level of resistance was so great that the aminoglycosides were virtually useless.^18^ The most common mechanism of resistance to aminoglycosides is enzymatic modification. These enzymes modify aminoglycosides at different –OH or –NH_2_ groups on the deoxystreptamine nucleus or on sugar moieties. The number of aminoglycoside-modifying enzymes identified to date, as well as the genetic environments in which the coding genes are found, are impressive.^19,20^ Moreover, aminoglycoside resistance can be supported by virtually any bacterium. Along with developing aminoglycosides resistant to as many modifying enzymes as possible, the following main strategies are being pursued for overcoming aminoglycoside modifying enzyme action: inhibiting enzyme action or expressing enzymes that modify enzyme action.

### 2.2. Blockers: modify the targets of antimicrobial agents

#### 2.2.1 Penicillin binding proteins

Penicillin-binding proteins (PBPs), membrane-associated macromolecules that are involved in cell wall synthesis and are targets of β-lactam antibiotics, have been exploited. However, PBPs are inactivated once they are acylated by β-lactam antibiotics, they cannot catalyze the hydrolysis of covalent acyl enzymes, they cannot perform the transpeptidation of peptidoglycans, and the cell wall is weakened. Despite the strength of β-lactam antibiotics, drug-resistant strains are becoming an increasingly serious issue around the world. The periplasm of Gram-negative bacteria such as *Pseudomonas aeruginosa* and *Escherichia coli* evades the action of β-lactamases. Another mechanism preventing antibiotics from reaching their macromolecular targets is to force them out of the bacterium *via* antibiotic efflux pumps, such as MexA and B-OprM pumps in *Pseudomonas* strains.^21^ PBPs that are highly mutated and resistant to drugs are also produced by some Gram-positive bacteria, such as streptococci that do not produce β-lactamases. A new, highly resistant class B PBP (PBP2a) has been acquired by *Staphylococcus aureus* (*S. aureus*) strains following horizontal transfer of mecA from a yet unidentified species.^22,23^ While resistant bacteria incorporate these key mutations in PBP, they still divide and turn over peptidoglycan at similar rates; there is a lack of knowledge about the mechanisms underlying this sustained catalytic efficiency.

#### 2.2.2 Processes of recycling peptidoglycan, the cell wall precursor

Bacterial cell walls contain large amounts of biopolymers that ensure the bacterial structural integrity to compensate for their inability to regulate osmotic pressure. In the cell wall, crosslinked peptidoglycan plays a major role, and an NAG-N-acetylmuramic acid (NAM) linear backbone is assembled. In this step, Lipid II is polymerized to yield a pentapeptide stem that is attached to NAM. In PBPs, typical peptide stems in G (−) bacteria are L-Ala-γ-D-Glu-*m*-DAP-D-Ala-D-Ala (in which DAP is diaminopimelate), which crosslink with other peptide stems on neighboring strands.^24^

As a dynamic matrix, the cell wall is constantly being constructed and recycled at the same time as enzymes are involved in the process. With each generation, approximately half of a G (−) bacteria's cell wall is remodeled.^25–27^ A fragment of the cell wall can be biologically recycled for cell wall synthesis, and bacteria as well as eukaryotes use them as messengers for communication. PBP4, a penicillin-binding protein with DD-carboxypeptidase and 4,3-endopeptidase activity, has emerged as a key player in the detection and response to β-lactam antibiotics.^28^ Muropeptides are also very relevant as signaling molecules that are capable of provoking the multiresistance protein AmpC. When *P. aeruginosa* is resistant to a β-lactam antibiotic, signaling molecules have been identified in only two peptides over 20.^29^ Antibiotic resistance can be caused by many mechanisms, including the expression of AmpC, but new mechanisms linking cell-wall recycling to resistance have been described recently.^30^ There is a metabolic pathway in *P. putida* that links cell wall recycling to an intrinsic resistance to phosphomycin. As peptidoglycan plays a crucial role in bacterial survival and is remodeled by a large number of proteins, an emerging pathway could link recycling and antibiotic resistance in the near future.

#### 2.2.3 Enzymes for DNA replication

An antimicrobial that inhibits bacterial DNA replication might be a promising strategy for combating antibiotic resistance because the replication of DNA involves multiple enzymes, and blocking any step in a single enzyme reaction can result in bacterial death, providing many potential targets for drug development; there is a conserved, distinct, or lack of expression of these enzymes in prokaryotic and eukaryotic cells, which confers selectivity to their corresponding inhibitors, preventing toxicity to humans.^31,32^ In clinics, only quinolones, which inhibit type II topoisomerases, are widely used,^33^ so any inhibitor of another DNA replication enzyme will provide a new antibacterial mechanism that is effective against a variety of antibiotic-resistant bacteria.

#### 2.2.4 Ribosomes

A number of mechanisms have evolved that allow bacteria to resist antibiotics, and a large part of these mechanisms use ribosomes as their target. Throughout eukaryotic cells, an essential process of protein synthesis is carried out by ribosomes. Ribosomes are among the most complex and conserved macromolecular machines in nature. Although ribosomes are large, most of the clinically relevant antibiotics target relatively few regions that are functionally important, which has led to the overlapping of many binding sites.^34,35^ There is overlap between PTC-targeting antibiotics and A-site tRNA binding sites, or the binding sites span both A- and P-sites. During translation, the PTC transports the growing polypeptide chain to the macrolides and SB classes by binding to their binding sites.^36–38^ Most peptide bonds are not prevented by macrolides or SB members *per se*, but they do alter peptide chain elongation, which results in a drop-off in peptidyl-tRNA and abortive translation, causing an imbalance in protein synthesis. A variety of ribosome-targeting antibiotics have been identified as having antibiotic-resistance mechanisms.^39–41^ How antibiotics become resistant is fairly well understood, as well as their clinical significance. In some cases, ribosome target sites can be mutated or modified to reduce or eliminate the ability to bind antibiotics. A variety of enzymes can degrade, modify, or excrete antibiotics directly from the cell. Thus, the intracellular concentration is reduced from toxic to nontoxic.

### 2.3. Expellers: pump out antimicrobial agents

#### 2.3.1 Efflux

One possible mechanism for multidrug resistance is the overexpression of efflux pumps. There are currently six family members of bacterial efflux pumps associated with efflux pathways. In one pump, ATP is directly used to drive transport *via* ATP-binding cassettes (ABCs). Five more types of active transporters utilize transmembrane gradients to capture electrochemical energy; these transporters are the major facilitator superfamily (MFS), the multidrug and toxin extrusion (MATE) family, the small multidrug resistance (SMR) family, the resistance-nodulation-cell division (RND) superfamily and the proteobacterial antimicrobial compound efflux (PACE) family.

The association between efflux and drug resistance was first described over 40 years ago. For drug resistance to be effective, there must be an equal or greater amount of efflux than influx. In turn, drug entry is determined by the composition of the cell envelope and the presence of porins or other entry channels. There is no doubt that porin loss can contribute to resistance, and it has been observed to occur during treatment.^42,43^ Additionally, several cases of efflux-mediated resistance have been reported among clinical isolates, some of which involve the overexpression of pumps.

It is essential to have a clear understanding of efflux regulation because the overexpression of efflux proteins is caused by alterations in the regulatory system and transcriptional regulator mutations, including local and global mutations.^44,45^ There can be differences in efflux regulation between species, as well as within species, based on the physiological state of the cell. There are many pumps that are interconnected and regulated by complex circuits. Inhibiting or deleting one pump can lead to the expression of others.^46–48^ This complicates the interpretation of resistant phenotypes caused by efflux pump networks.

### 2.4. Decreased intracellular concentration

#### 2.4.1 Decreased membrane permeability

Decreasing the degree of membrane permeability may increase the antibiotic resistance. By mediating the transport of molecules, the pores in the outer membrane play a critical role in the resistance and virulence of *Acinetobacter baumannii* (*A. baumannii*) strains. The decrease in membrane pore protein density of *Acinetobacter A. baumannii* was associated with increased carbapenem resistance.^49,50^ As another membrane porin involved in *A. baumannii*, OmpA has been reported to be resistant to aztreonam, chloramphenicol, and nalidixic acid. Studies have shown that OmpA increases virulence, lung infection, sepsis, and mortality.^51–53^

## 3. MDR modulators of natural product-derived compounds from plants

Even though bioactive molecules with plant origins are utilized for a variety of purposes, the use of these molecules for fighting MDR bacteria and restoring the effectiveness of antibiotics in clinics has largely been ignored. According to medicinal chemists, natural products are miscellaneous molecules that have evolved to interact with a wide variety of protein targets to accomplish specific goals.^54–57^ Hence, natural sources are considered to be an advantageous source for discovering new antimicrobial molecules. Over half of the drugs approved by the American Food and Drug Administration (FDA) are based on or inspired by natural products.

The necessary starting materials for drug discovery that are used by pharmaceutical companies have always been provided by nature, since these materials have always solved many complex clinical problems. Natural products have been studied extensively as modulators of MDRs. The MDR modulators of natural product-derived compounds from plants are summarized in Table 1 as three major categories: flavonoids, alkaloids and terpenoids.

**Table 1:** Natural product-derived compounds from plants as multidrug resistance modulators in microorganisms^a^

Classification	Compound	Source	Antibacterial activity	Mechanism	Reference
Flavonoids	(1) Isobavachalcone (IBC)	Psoralen	MRSA and VRE (MIC_50_: 0.5 μg mL; MIC_90_: 4–8 μg mL^−1^)	Membrane homeostasis	66
	(2) α-Mangostin (AMG)				
	Quercetin	—	*P. aeruginosa* and *A. baumannii* (MIC: 16–256 μg mL^−1^)	Enzyme inhibition and efflux pump inhibition	67
			ATCC 25922 (MIC: 1 μg mL^−1^)		
			*E. coli* clinical isolates (MIC: 64 μg mL^−1^)		
	(1) 3,7-Diacylquercetin	Quercetin	Multidrug-resistant gram-positive (MIC: 0.13 to 128 μg mL^−1^)	Enzymatic inhibition (DNA gyrase and topo IV)	69
	(2) Quercetin 6′′-acylgalactoside				
	(3) Quercetin 2′′,6′′-diacylgalactoside analogues				
	(1) Procyanidin B3-3-*O*-gallate	*W. uniflora*	MRSA	Abnormal cell formation	71
	(2) Rhamnetin 3-*O*-(6′′-galloyl)-β-d-glucopyranoside				
	(3) Rhamnetin 3-*O*-α-l-rhamnopyranoside				
	(4) Quercetin 3-*O*-(6′′-galloyl)-β-d-glucopyranoside				
	(1) Isobavachalcone;	—	*E. coli* AG100A and *E. aerogenes* EA298 isobavachalcone (MIC: 8 μg mL^−1^)	Efflux pump components (AcrAB, TolC)	72
	(2) Diospyrone		Diospyrone (MIC: 4 μg mL^−1^)		
	Lupinifolin	*Albizia myriophylla*	MDR enterococcal clinical isolates (MIC: 0.5 and 2.0 μg mL^−1^)	Membrane permeability and salt tolerance	73
	Phloretin	Apple	*E. coli* ZJ478 or *Salmonella* sp. Stain (MIC: 2 μg mL^−1^)	Cell membranes	74–76
	Type A procyanidin (TAP	*Cinnamomum zeylanicum*	UPEC	Biofilm formation	77
	Artonin I	*Morus mesozygia Stapf*	MDR bacterial (MIC: 4–8 mg L^−1^)	Efflux pump and cell membrane	78
	AFPO	Chalcones	*S. aureus* 10 strain (MIC: 1024 μg mL^−1^)	Internal resistance mechanism	79
			*E. coli* 06 strain (MIC: 256 μg mL^−1^)		
	3′,4′,7-trihydroxyflavone	Kernel	*P. stuartii* ATCC299645 (MIC: 4 μg mL^−1^)	Efflux pumps	80
	Epicatechin gallate (ECg)	Green tea	*S. aureus* BB568, EMRSA-15, EMRSA-16 (MIC: 128 mg L^−1^)	Cell membrane	89
	Curcumin	—	*A. baumannii* (MIC >256 μg mL^−1^	—	65
	Epigallocatechin gallate	Green tea	*A. baumannii* (MIC >128–1024 μg mL^−1^)	—	65
	Eriodictyol	Citrus fruits	*S. aureus* USA 300 (MIC >512 μg mL^−1^)	Inhibitor of SrtA	92
	Rutin	—	*K. pneumoniae* ATCC700603 (MIC: 1024 μg mL^−1^)	Downregulated *luxS* gene and wabG gene	63
			*E. coli* ATCC25922 (MIC: 512 μg mL^−1^)		
	Baicalein	—	MRSA	Cell wall and Tet K-mediated tetracycline efflux	70
	Ursolic acid (UA)	Apple pomace	CRKP (MIC: 0.8 mg mL^−1^)	Cell membrane integrity	93
	5-Hydroxy-3,7,4′-trimethoxyflflavone	V. *gardneriana*	MDR bacterial strains *S. aureus* 358 and *E. coli* 27 (MIC≤512 μg mL^−1^)	—	94
Alkaloids	Compound 1f (with a 7-phenyl group)	7-Substituted cyclophosphamide (CBBR)	MRSA and VRE (MIC: 1-8 μg mL^−1^)	DNA Topo IV ParE subunit	102
	1,4-Naphthoquinones	—	MRSA (MIC: 0.0078 to 0.125 mg mL^−1^)	—	103
	Chelerythrine (CHE)	—	CRSM (MIC: 125 mg mL^−1^)	Integrity of the cell membrane	105
	Berberine hydrochloride (BBH)	Berberine	MDR *A. baumannii* (MIC: 256 mg L^−1^)	AdeB and AdeB transporters	106
	Piperine	White pepper	MDR strains of *P. aeruginosa, E. coli* (in the presence of 125 mg mL^−1^)	—	107
	Squalamine	Dogfish shark squalus acanthias	*P. aeruginosa* ATCC 27853 (MIC: 8 mg L^−1^)	Cell membranes	108
			*E. coli* ATCC 25922 (MIC: 4 mg L^−1^)		
			*S. aureus* ATCC 25923 (MIC: 2 mg L^−1^)		
	*N*-tetradecyl derivative (BnI-14)	Quaternary ammonium compounds (QACs)	*S. aureus* ATCC 29213 (MIC: 0.03 μg mL^−1^)	Membrane disruption and DNA	110
			*S. aureus* ATCC 29213 clinical/MRSA (MIC: 0.12 μg mL^−1^)		
			*E. coli* clinical (MIC: 0.98 μg mL^−1^)		
	Arborinine	*R. angustifolia*	*Candida albicans* arborinine (MIC: 250 μg mL^−1^)	ICL1 gene	111
			Arborinine (MIC: 500 μg mL^−1^)		
	Catharanthine	*Catharanthus roseous*	*P. aeruginosa* (MIC: 400 mg L^−1^)	Efflux pump proteins MexA, MexB and OprM	113
	Chanoclavine	*I. muricata*	*E. coli*	Efflux pumps and the cell envelope	115
Terpenoids	Limonene	—	*E. coli* (MIC: 16 μg mL^−1^)	Cell membrane and DNA transcription and translation	118 and 119
	Carvacrol	—	*S. aureus* (MIC: 256 μg mL^−1^)	Efflux pump inhibition	120 and 122
	Thymol				
	Oleanolic acid	—	*E. faecalis* (MIC: 6.25 mg L^−1^)	Efflux pump	121
			MRSA COL_OXA_ (MIC: 1600 mg L^−1^)		
	Clerodane diterpene 16α-hydroxycleroda-3,13(14)-Z-dien-15,16-olide (CD)	*Polyalthia longifolia*	MRSA-ST1745/ST2071/P4620 (MIC: 31.25 μg mL^−1^)	EtBr efflux	123
			MRSA-P4627/P4423/ST3151 (MIC: 15.62 μg mL^−1^)		
	Geraniol (Ger)	Geranium oil	*C. albicans* (MIC: 225 μg mL^−1^)	Cell wall integrity and cell adhesion	124
	Oleanane triterpenoid aegicerin	*ClaVija procera*	MTB (MIC: 1.6-3.12 μg mL^−1^)	—	125
	Artesunate (ART)	*Artemisia annua L*	MDR *E. coli* isolates	mRNA expression of qnrB and qnrS	126
	Glycyrrhizin (GLY)	*Glycyrrhiza glabra*	MDR9 (MIC: 40 mg mL^−1^)	—	127
			B1045 (MIC: 15 mg mL^−1^)		
	Conjugates	—	*S. aureus* (MIC: 20 μg mL^−1^)	—	129
			*E. coli* (MIC: 20 μg mL^−1^)		
	1,8-Cineole	Rosemary volatile oil	*E. coli*	Biofilm biomass disruption	135

a MRSA, methicillin-resistant *Staphylococcus aureus*; VER, vancomycin-resistant *Enterococcus*; MIC_90_, lowest drug concentration inhibiting 90% of bacterial or fungi growth; MIC, minimum inhibitory concentration; MDR, multidrug-resistant; UPEC, Uropathogenic *Escherichia coli*; CRKP, carbapenem-resistant *Klebsiella pneumoniae*; CRSM, carbapenem-resistant *Serratia marcescens*.

### 3.1. Flavonoids

**Fig. 2:**
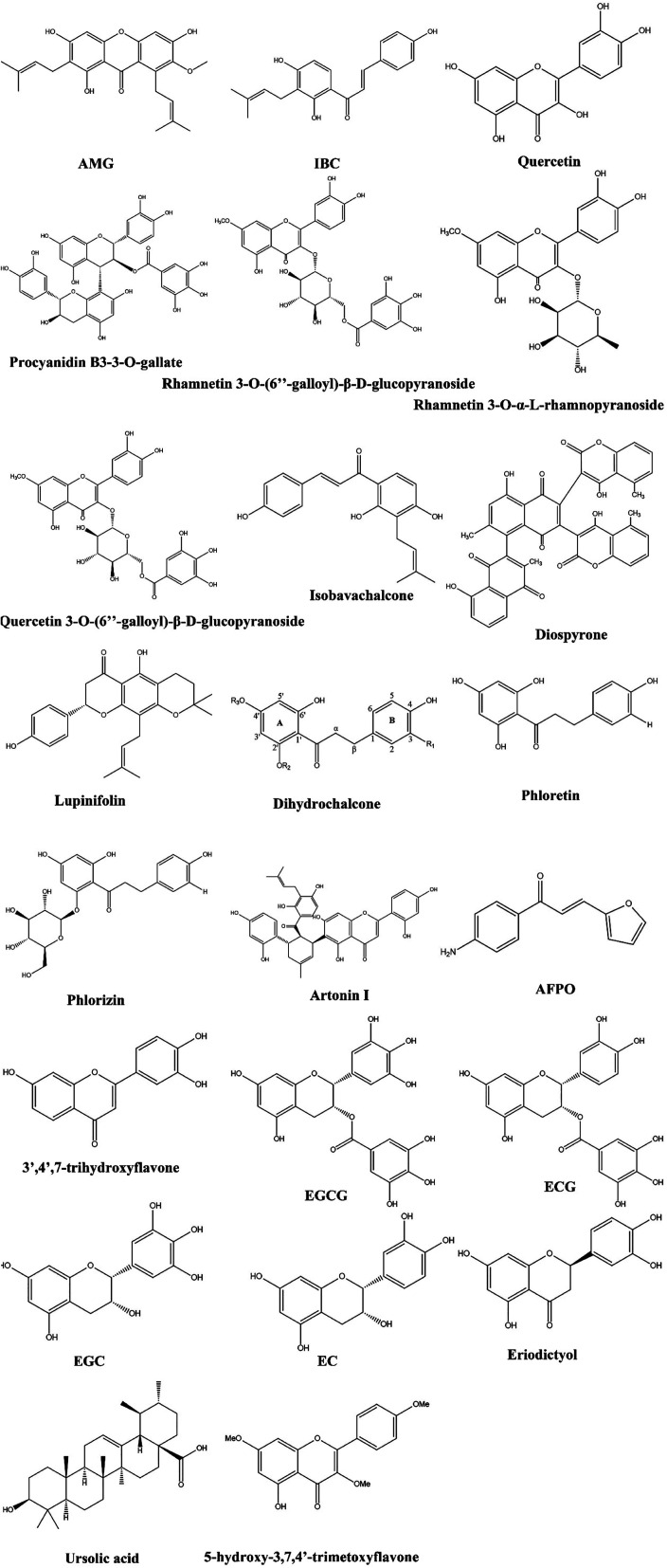
Structure of flavonoids having MDR modulatory activity.

Among the most important classes of phenolic compounds are flavonoids, which are plant secondary metabolites in nonglycosylated (aglycones) or glycosidic states. In addition to the 2-phenylchromane ring system, flavonoids also have different subclasses depending on the substitution at ring B and oxidation status of ring C.^58,59^ There has been considerable evidence that flavonoids possess a variety of pharmacological properties, including anticancer, anti-inflammatory, and antiviral properties.^60–62^ Additionally, flavonoids have been found to exhibit antibacterial properties against many pathogenic microorganisms, such as *Staphylococcus aureus* (*S. aureus*), *Vibrio harveyi*, *Pseudomonas aeruginosa* (*P. aeruginosa*), and *Enterococcus faecalis* (*E. faecalis*).^63–65^ There are over 8000 known flavonoids in plants, making them a potentially valuable resource for new antibiotic discoveries. We reviewed the potential antimicrobial activity of flavonoids against fungal and bacterial infections in Sections 3.1.1–3.1.13, and their structure was drown in Fig. 2.

#### 3.1.1 Isobavachalcone (IBC) and α-mangostin (AMG)

Analyses of structure-activity relationships show that prenylation modulates their activity in flavonoids and produces two compounds, isobavachalcone (IBC) and α-mangostin (AMG). Additionally, AMG and IBC are bactericidal against Gram-positive bacteria and restore the susceptibility of colistin against Gram-negative bacteria. AMG and IBC had MIC_50_ values of 0.5 μg mL^−1^ and MIC_90_ values of 4–8 μg mL^−1^ against methicillin-resistant *Staphylococcus aureus* (MRSA) and vancomycin-resistant enterococcus (VRE), respectively. AMG and IBC are capable of targeting almost all kinds of phospholipids in bacterial membranes and involve faster time-killing dynamics than that of nearly all antibiotics currently used in clinics, including vancomycin. Despite their ability to disrupt membranes, these agents are unlikely to develop resistance to antibiotics, since bacteria are unable to change their membranes without losing functions.^66^ In mice, AMG or IBC single-dose models were constructed for wound infection and intestinal colonization of *S. aureus*. The results showed that by using AMG and IBC, it may be possible to reduce the number of foodborne bacterial infections and poisonings.

#### 3.1.2 Quercetin

In 2002, Denny *et al.* demonstrated for the first time that quercetin inhibited L1 metallo-β-lactamase.^67^ At high concentrations, quercetin has been found to inhibit Gram-positive and Gram-negative bacterial growth. By inhibiting blaNDM expression, quercetin could potentiate meropenem activity. The combination of quercetin-meropenem induced bacterial death by altering cell morphology and disrupting cell wall/membrane integrity, inhibiting carbapenem hydrolysis in periplasmic spaces and preventing bacterial efflux. Furthermore, quercetin inhibits carbapenemase and efflux pump activity in carbapenem-resistant *E. coli*, *Klebsiella pneumoniae*, *P. aeruginosa* and *A. baumannii*.^68^ Das *et al.* (2019) identified naphthoate synthase (1,4-dihydroxy-2-naphthoyl-CoA synthase, EC: 4.1.3.36; DHNS, *Ef*DHNS) as an anti-*E. faecalis* target in novel antibacterial drugs. Moreover, quercetin inhibits *Ef*DHNS catalytic activity, potentially allowing the use of quercetin for the treatment of MDR *E. faecalis* infections.^69^ According to structure-guided molecular design, quercetin diacylglycoside analogs were optimized, including 3,7-diacylquercetin, quercetin 6′′-acylgalactoside, and quercetin 2′′,6′′-diacylgalactoside analogs, which exhibited four times greater activity against Gram-positive bacteria than that of previous compounds. A possible mechanism of action of this drug is to inhibit bacterial DNA gyrase and topoIV enzymes simultaneously. More importantly, its acute toxicity in mice was very low, as well as realistic *in situ* intestinal absorption in rats.^70^ In another study, quercetin significantly enhanced the tetracycline sensitivity of *E. coli*. According to further studies, the combination therapy causes profound ultrastructural changes in the cells that result in an increase in permeability and a weakening of the cell envelope.^71^

#### 3.1.3 The methanolic extract of *W. Uniflora*

Based on phytochemical analysis of the methanolic extract of *W. uniflora* leaves coupled with LC/MS analysis, the following flavonoid analogs (1–4) were isolated: procyanidin B3-3-*O*-gallate (1), rhamnetin 3-*O*-(6′′-galloyl)- β-d-glucopyranoside (2), rhamnetin 3-*O*-α-l-rhamnopyranoside (3), and quercetin 3-*O*-(6′′-galloyl)-β-d-glucopyranoside (4). In addition to inhibiting MRSA biofilm formation, these compounds also synergize with methicillin. Compounds achieved this synergistic effect by remodeling metabolism, and defects in central carbon metabolism and glutamine biosynthesis led to abnormal MRSA cell formation and a reduction in biofilm formation. Moreover, rhamnetin 3-*O*-(6′′-galloyl)-β-d-glucopyranoside (2) and quercetin 3-*O*-(6′′-galloyl)-β-d-glucopyranoside (4) showed no significant cytotoxicity.^72^

#### 3.1.4 Isobavachalcone and diospyrone

Isobavachalcone and diospyrone were evaluated against multidrug-resistant (MDR) Gram-negative bacteria. The results showed that both compounds had intrinsic antibacterial properties against Gram-negative bacteria, and in the presence of an efflux pump inhibitor, their activity was significantly enhanced (MIC values decreased to below 10 μg mL^−1^). A significant increase in activity was also observed for diospyrone, and isobavachalcone was shown to be efficient against strains that have deletions of major efflux pump components (AcrAB, TolC). As a result of the study, it appears that isobavachalcone and diospyrone could be used as new antimicrobials against MDR bacteria, particularly by combining them with efflux pump inhibitors, which reinforces their activity.^73^

#### 3.1.5 Lupinifolin

Lupinifolin, a prenylated flavonoid extracted from *Albizia myriophylla Benth.*, was shown to exhibit a potent antimicrobial effect on enterococci. The bacterial membrane is damaged by lupinifolin, which inhibits *S. aureus* and *Streptococcus* mutans growth. Significantly reducing the salt tolerance of lupinifolin-disrupted bacterial cell membranes can also cause the membranes to lose their ability to osmoregulate effectively or to exclude toxic materials, eventually causing acterial cell death. As lupinifolin penetrated and passed through the cell wall of *S. mutans*, lupinifolin was observed in the cytoplasm of lupinifolin-treated cells. By doing so, lupinifolin disrupts the integrity of the cytoplasmic membrane, resulting in the death of the bacteria. Research on lupinifolin's antibacterial activity against a variety of MDR enterococci clinical isolates has furthered our understanding of its properties and mechanisms.^74^ In light of this, lupinifolin could play a critical role in fighting MDR enterococcal infections. There is potential for this compound to be developed into a new potent therapeutic agent.

#### 3.1.6 Dihydrochalcone

Apples contain a specific group of flavonoids called dihydrochalcones, which possess a chemical structure of C6–C3–C6, and there is no direct link between the A-ring and B-ring; instead, a flexible C3 chain connects them. It was found that dihydrochalcones exhibited excellent antibacterial properties against both Gram-positive and Gram-negative bacteria. The glycosylation of the A-ring at the 2′-position, as well as the hydroxyl group at the 3-position of the B-ring, was important for the antibacterial activity of dihydrochalcones. A positive correlation might exist between dihydrochalcone hydrophobicity and its antibacterial properties. Dihydrochalcones act mainly by damaging cell membranes in antibacterial mechanisms.^75^ Despite this, strain type also influenced antibacterial mechanisms. The most abundant dihydrochalcone compound in apple is phlorizin, which accounts for 60–90% of the overall flavonoids. Gram-positive bacteria were inhibited by phloretin, which exhibited the highest levels of antimicrobial activity, especially *S. aureus* ATCC 6538, *L. monocytogenes* ATCC 13932, methicillin-resistant *S. aureus* clinical strains and *S. typhimurium* ATCC 13311. Considering that C20 contains a free hydroxyl group, it can be assumed that glycosyl molecules, or their absence, play an important role in interactions with other biological molecules, and C20, C30, and C50 glycosylated structures demonstrate dramatic decreases in antimicrobial activity. In addition, this compound's antimicrobial activity can be understood through the analysis of cytosolic enzymes from *S. aureus*. Essentially, phloretin inhibits *S. aureus* energetic metabolism by lowering lactate dehydrogenase (LDH) and isocitrate dehydrogenase (IDH) enzyme activity. It significantly inhibits catalase activity and determines the use of biological fuels (sugars, fatty acids, amino acids) by bacteria and its ability to deal with oxidative damage.^76^ Phloretin restored polymyxin E's sensitivity to *E. coli* ZJ478 or *Salmonella* sp. stain HYM2 from 64 μg mL^−1^ to 2 μg mL^−1^ for both bacteria. Phloretin and polymyxin E were used to treat a variety of strains, including those that were mCR-1 positive and negative, and the fractional inhibitory concentration (FIC) values were all determined to be under 0.5. Despite this, bacterial resistance was not triggered by the combination of phloretin and polymyxin E. Combined treatment led to an 80% survival rate of infected mice, as well as a significant decrease in cecal colony counts.^77^

#### 3.1.7 Type A procyanidin

The antimicrobial activity of Type A procyanidin (TAP) from *Cinnamomum zeylanicum* against QSLUPEC7 (a multidrug-resistant strain that forms strong biofilms) was researched. In contrast to the growth of the MDR strain, TAP treatment affected the formation of biofilms (∼70%). Furthermore, different pH levels were used to study the synergy between TAP and nitrofurantoin (NIT).^78^ The results of the study indicate that depending on the pH level, TAP interacts synergistically with nitrofurantoin. At pH 5.8, the greatest growth inhibition was observed. Gene expression analysis indicated that TAP inhibits *Uropathogenic Escherichia coli* (UPEC) fimbriae adhesins alone or in combination with NIT. In conclusion, TAP inhibits the growth of multidrug-resistant strains of UPEC without affecting their antibiofilm activity.

#### 3.1.8 Artonin I

Artonin I inhibits MDR *S. aureus* growth both on its own and when combined with different antibiotics. Artonin I also showed potential to reverse resistance in further mechanistic studies. Depolarization of bacterial cell membranes and inhibition of efflux mechanisms have been revealed by flow cytometric studies.^79^ Artonin I is responsible for the production of ROS, which causes cell death. Artonin I damages cell membranes and alters the pattern of cell division, resulting in cellular destruction.

#### 3.1.9 AFPO

The chalcone (*E*)-1-(4-aminophenyl)-3-(furan-2-yl)-prop-2-en-1-one (C_13_H_11_NO_2_) AFPO has altered the effectiveness of norfloxacin, penicillin, ampicillin/sulbactam, and gentamicin against multiresistant strains of *S. aureus*, combining all antibiotics synergistically and reducing concentration requirements. AFPO also modified the activities of norfloxacin, gentamicin, and penicillin and lowered the MIC for the *E. coli* 06 strain.^80^ This means that AFPO in combination with antibiotics tested may reduce the effects of medications used to treat infections caused by *S. aureus* 10 and *E. coli* 06 in humans.

#### 3.1.10 3′,4′,7-trihydroxyflavone

Kernel methanol extract (MFS) and one of its derived compounds, 3′,4′,7-trihydroxyflavone, exhibit important antibacterial activity against MDR strains. On the tested MDR, a crude MFS extract and 3′,4′,7-trihydroxyflavone both showed improved antibacterial activity in the presence of PAβN (EPI) on 11/15 (73,37%) and 7/7 (100%), respectively.^81^ Based on these results, we believe that crude MFS extracts and their active constituents may be substrates for efflux pumps and thus have an intracellular target. 3′,4′,7-Trihydroxyflavone enhanced antibiotic activity in most bacterial strains.

#### 3.1.11 Tea polyphenols

In addition to having numerous biological activities, tea always attracts worldwide interest. Due to their ability to scavenge free radicals, tea polyphenols are well known for their medicinal value, and several studies have shown that they can improve the body's immune system and alleviate a number of symptoms.^82,83^ In addition, there is much evidence that tea catechins have antiviral and antimicrobial properties. *In vitro* anti-methicillin resistant *Staphylococcus aureus* (MRSA) activity can be enhanced by combining several β-lactams with Japanese green tea extracts. Additionally, green tea extract itself was shown to inhibit *S. aureus* ATCC 25923 as well as MRSA at 400 μg mL^−1^ MICs.^84^ As much as 30-40% of the water-soluble solids and 20–30% of the dry matter in green tea are flavan-3-ols (also called catechins). Catechins in green tea mainly include ester (−)-epigallocatechin-3-*O*-gallate (EGCG), epicatechin-3-*O*-gallate (ECG), (−)-epigallocatechin (EGC), (−)-epicatechin (EC) together with some (−)-gallocatechin-3-O gallate (GCG), and (−)-catechin-3-*O*-gallate (CG).^85,86^ In total, EGCG accounted for nearly 59%, followed by EGC at 19%, ECG at 13.6%, and EC at 6.4%. Studies have shown that green tea catechins, mainly EGC, EGCG, and ECG, have antibacterial properties.^87,88^ Natural and semisynthetic catechins, as well as their gallated derivatives, were tested for anti-MRSA activity, and it was found that they have MICs in the range of 64–256 mg L^−1^.^89^ Gallate esterification at the C-ring of catechins is also a method of enhancing oxacillin and lactam antimicrobial activity. The use of various 3-*O*-acyl chains instead of the gallate moiety of epicatechin gallate (ECg), could also enhance the antibacterial strength of catechins against MRSA.^90^

A high percentage of therapeutic antimicrobials are no longer effective against MDR strains, and EGCG should be able to improve their treatment. Another study found that EGCG was effective in modifying resistance in *Campylobacter* spp. by inhibiting efflux pump activity in the wild-type strain NCTC 11168 as well as in the cmeB, cmeF, and cmeR mutants.^91^ EGCG prevents conjugative drug resistance by reducing the transfer of drug resistance plasmids in a dose-dependent manner. Tetracycline resistance is reversed by EGCG when it inhibits the Tet(K) efflux pump (EP) in *staphylococci*, resulting in antibiotic sensitivity in infected staphylococci.^92^

Curcumin (CCM) and EGCG were investigated and evaluated against multidrug-resistant strains of *A. baumannii via* checkerboard and time-kill assays. CCM had an MIC of >256 μg mL^−1^ against all strains of *A. baumannii*, whereas EGCG had an MIC of 128–1024 μg mL^−1^. Five of nine isolates showed synergy, and four isolates showed additive effects, according to checkerboard studies. CCM's MIC was reduced by 3- to 7-fold when EGCG was added, and a CCM MIC of 4 μg mL^−1^ was the strongest interaction. In comparison with the most effective polyphenol alone, a combination of CCM and EGCG (1 : 8 and 1 : 4) demonstrated 4- to 5-log reductions in viable counts after 24 hours. Although CCM alone exhibits little antibacterial activity, EGCG significantly enhances its activity against MDR *A. baumannii*. Combinations of these two substances may be useful for medicinally treating or preventing infections caused by *A. baumannii*.^65^

#### 3.1.12 Eriodictyol

Eriodictyol, a reversible inhibitor of SrtA with an IC50 of 2.229 ± 0.014 g mL^−1^, can be used as an innovative tool for countering resistance as well as virulence. Eriodictyol inhibited bacterial adhesion to fibrinogen and decreased biofilm formation and staphylococcal protein A (SpA) anchoring to cell walls. It was found that eriodictyol and SrtA had a strong interaction during fluorescence quenching experiments. In subsequent mechanistic studies, eriodictyol was shown to interact with the R197 amino acid residue of SrtA. Moreover, eriodictyol inhibited adhesion-dependent invasion of A549 cells by *S. aureus* and was effective in treating mouse pneumonia.^93^

#### 3.1.13 Other flavonoids

Rutin, a flavonoid, induces luxS and wabG gene expression to inhibit *K. pneumoniae* in biofilm cells. A positive correlation was observed between biofilm biomass and the type III fimbriae biosynthesis gene mrkA.^63^

Through its direct binding to the peptidoglycan in the cell wall, baicalein can work synergistically with tetracycline to compromise the integrity of the cell wall. In addition to inhibiting Tet K-mediated tetracycline efflux, baicalein inhibits Tet M and other pumps that are responsible for methicillin-resistant *S. aureus* (MRSA) growth.^71^

An antimicrobial agent found in apple pomace, ursolic acid (UA), exhibits antimicrobial properties against some microorganisms. UA was found to be effective against *Klebsiella pneumoniae* (CRKP) at an MIC of 0.8 mg mL^−1^. The drug disrupted CRKP's cell membrane integrity, inhibited biofilm formation, and inactivated cells that were encased in biofilms.^94^

The compound 5-hydroxy-3,7,4′-trimetoxyflavone, which has a chemical formula of C_18_H_16_O_6_, was isolated from the leaves of *V. gardneriana*. In combination with norfloxacin and gentamicin, this flavone exhibits antimicrobial activity against the MDR *bacteria S.* aureus 358 and *E. coli* 27. Hence, this natural compound contributes to the control of resistant bacteria by increasing antibiotic activity.^95^

### 3.2. Alkaloids

**Fig. 3:**
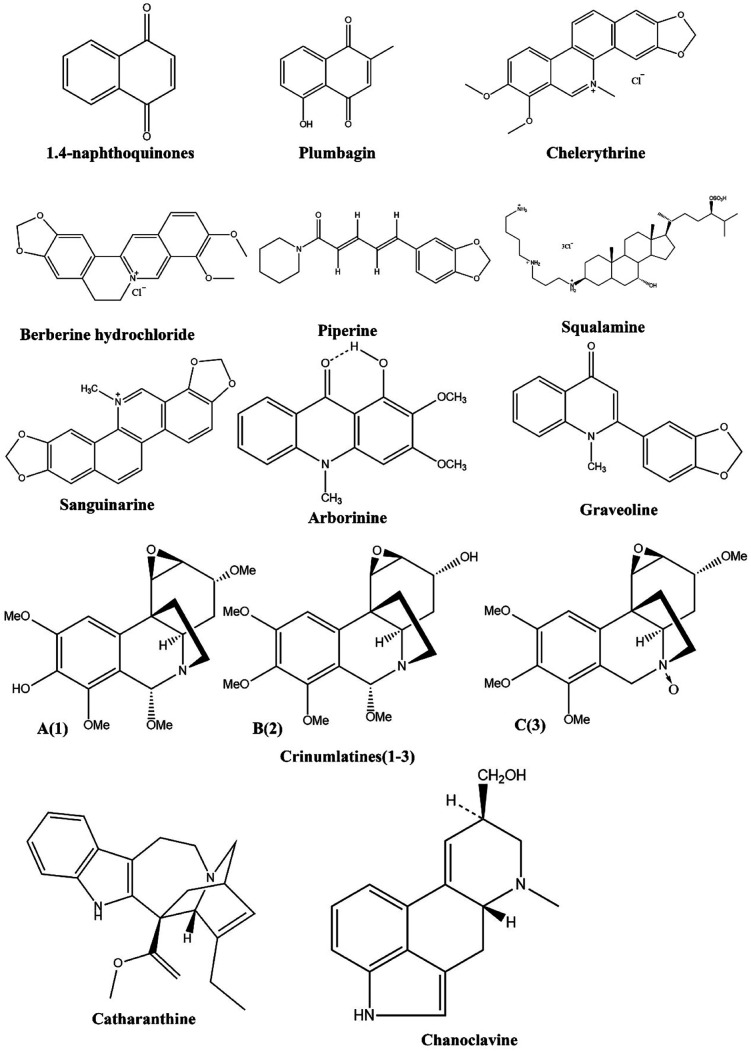
Structure of alkaloids having MDR modulatory activity.

Compared to synthetic antibacterial agents, alkaloids have fewer side effects, a higher efficacy and superior antibacterial properties against both resistance acquired pathogens and nonresistance acquired pathogens.^96,97^ In addition, alkaloids combined with antibiotics increased their efficacy, reduced side effects, and increased their concentration.^98–100^ In addition to inhibiting protein synthesis and efflux pumps, alkaloids inhibit the formation of biofilms, inhibit drug deactivating enzymes, and destroy cell membranes and cell walls.^101,102^ Using this natural gift to its full potential requires advanced studies. A variety of advanced methods, such as virtual screening, molecular modeling, and biological studies, can be used to alter the properties of these substances. This new era of antimicrobial agents may arise through the development of these alkaloids (Fig. 3).

#### 3.2.1 Cycloberberine (CBBR)

**Fig. 4:**
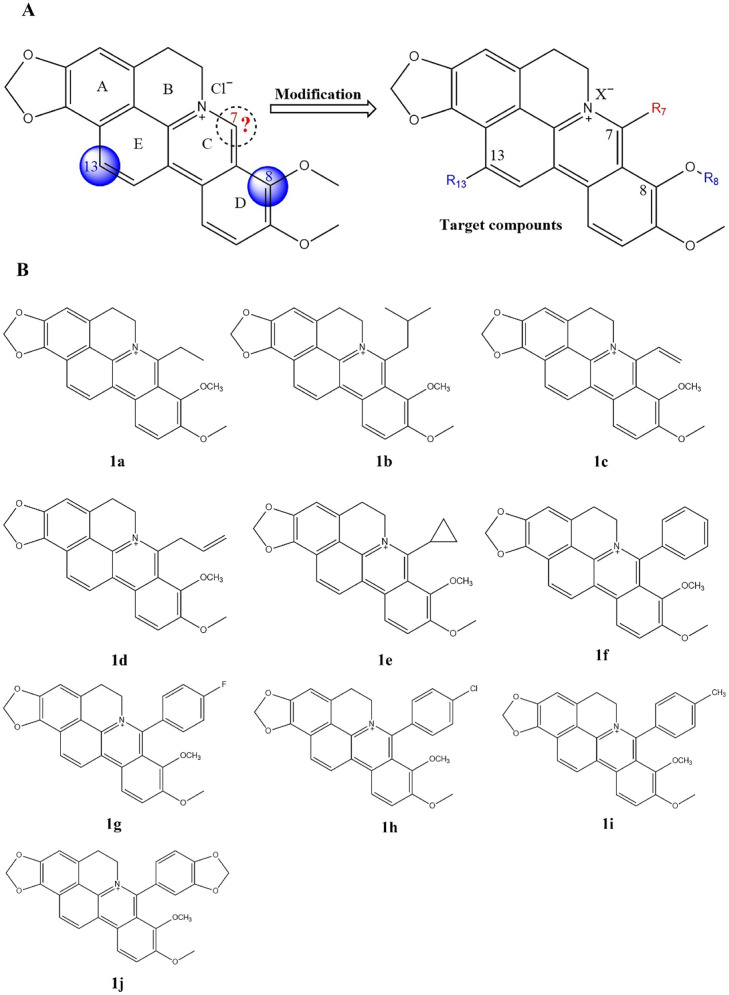
(A) The chemical structure of CBBR and the structure modification strategy. (B.) the structure of the target compounds 1a–j.

A novel family of MRSA agents has been identified using cycloberberine (CBBR) scaffolds that were constructed from Chinese natural *alkaline berberine* (BBR). Based on the structure-activity relationship (SAR), this compound series was significantly more potent against both reference strains and MDR clinical isolates by substituting at C8 or C13 (Fig. 4A). CBBR 7-monosubstitution was the focus of the initial SAR study. After attaching aliphatic chains or rings to the C7 position, compounds 1a–e (Fig. 4B) (with MIC values between 2 and 64 μg mL^−1^) display moderate antimicrobial activity against all tested strains, which is better than CBBR. Furthermore, measurements were performed on compounds 1f–j (Fig. 4B) with different substituted benzene groups, and compound 1f showed promising activity against both MRSA and vancomycin-resistant *Enterococcus* (VRE) strains. In contrast to vancomycin, compound 1f with a 7-phenyl group shows higher activity against MRSA and VRE, with MIC values of 1–8 μg mL^−1^. Based on its rapid bactericidal action against MRSA, 1f might target the DNA ParE subunits of Topo IV bacteria, suggesting that antibacterial drugs currently used have a different mode of action. Compared with SAR results at the C8 or C13 positions, a phenyl ring at the C7 position may enhance the antimicrobial spectrum. Further studies indicated that antimicrobial activity was dependent on the methylenedioxy ring.^103^

#### 3.2.2 1,4-Naphthoquinones

It was demonstrated that 1,4-naphthoquinones inhibit bacterial growth. In both Gram-positive and Gram-negative bacteria, bacteriostatic and bactericidal effects are observed with 1,4-naphthoquinone and plumbagin. In clinical strains of methicillin-sensitive *S. aureus* and MRSA, 1,4-naphthoquinone showed synergistic interactions with imipenem, cefuroxime, and cefotaxime. In MRSA cultured with ATCC, 1,4-naphthoquinone and cefotaxime have synergism. Combining 1,4-naphthoquinone with imipenem produced only an additive effect, while 1,4-naphthoquinone and cefuroxime had an antagonistic effect. While 1,4-naphthoquinone on its own is not as effective as common antibiotics, it synergizes with imipenem, cefuroxime, and cefotaxime in the treatment of MRSA, and it may be used as an adjunctive antibiotic to treat multidrug resistant bacteria.^104^

#### 3.2.3 Chelerythrine (CHE)

The natural product chelerythrine (CHE) is a benzophenanthridine alkaloid with antimicrobial potential. In the case of methicillin-resistant *Staphylococcus aureus* and *E. coli* producing extended-spectrum β-lactamases, CHE greatly improved the antibacterial efficacy of the antibiotics.^105^ CHE had an MIC of 125 mg mL^−1^ against CRSM, inhibited growth and destroyed the integrity of the cell membrane, and clearly altered the morphology of the cell. Biofilm components are produced by sub-MIC CHEs, which showed robust inhibitory effects against CRSM biofilm formation. In addition, according to CLSM- and FESEM-mediated evaluation of biofilm damage and biofilm persistence, biofilms can be compromised by CHE at high concentrations and remove previously formed biofilms.^106^

#### 3.2.4 Berberine hydrochloride (BBH)

Berberine hydrochloride (BBH) is the most common form of berberine. An *in vitro* study was conducted to determine the synergistic effects of BBH with antibiotics against MDR *A. baumannii*. There was weak antimicrobial activity for BBH alone against MDR *A. baumannii* (MIC of 256 mg L^−1^). A dramatic increase in susceptibility to antimicrobials when fractional inhibitory concentration index values <0.5 was achieved, even reversing the resistance of MDR strains. According to an *in vivo* study, BBH with sulbactam had greater antimicrobial effectiveness than that of monotherapy in neutropenic murine thigh infections. According to the antibiotic-sensitizing mechanism, boosting the expression of AdeB and binding to AdeB transporters resulted in a lower uptake of BBH, resulting in fewer antibiotics being extruded by the AdeABC pump. As a result of knockout of the adeB gene, BBH was more readily taken up by MDR strains, and resistance to antibiotics as well as synergistic effects between antibiotics and BBH were lessened. As a result, BBH effectively resensitizes this MDR pathogen to antibiotics that have become almost ineffective due to antibiotic resistance.^107^ Due to this, BBH may work synergistically with antibiotics and have antibiotic-sensitizing activity against MDR *A. baumannii*.

#### 3.2.5 Piperine

The growth of *V. cholerae*, an active ingredient of white pepper, with 200 and 300 μg mL^−1^ piperine was inhibited regardless of biotypes or serogroups. Piperine (in the presence of 200 μg mL^−1^) also showed growth inhibitory activity against MDR Gram-negative bacteria, including toxigenic *Vibrio cholerae*, *Pseudomonas aeruginosa* and enterohemorrhagic/enteroaggregative *E. coli* O104 German outbreak strains.^108^

#### 3.2.6 Squalamine

Squalamine, a natural aminosterol, has been demonstrated to possess antibacterial properties against several drug-resistant bacteria and molds. A squalamine's positively charged amino group interacts with a Gram-negative bacteria's lipopolysaccharide's negatively charged phosphate groups. As a result of its depolarizing effect, it causes cell death in Gram-positive bacteria, similar to colistin (polymyxin drug). Colistin displaces the divalent cations (Ca^2+^ and Mg^2+^) after forming a bond with LPS's phosphate groups.^109^

#### 3.2.7 Other alkaloids

*Chelerythrine*, an isoquinoline alkaloid, inhibits cellular division and nucleic acid synthesis. A protein called FtsZ, which contributes to Z ring formation during cell division, is tampered with by isoquinoline, inhibiting cellular division.^110^ The mechanism of action of quaternary ammonium compounds (QACs) targets the bacterial membrane, making them effective antimicrobial agents. For N-benzylimidazole, the synthesis of QACs based on imidazole derivatives, the activity varied in the range of the lowest inhibitory concentration (MICs) to 7 ng mL^−1^. A bacterial biofilm-fighting compound, *n*-tetradecyl derivative (BnI-14), was also found to bind to DNA, implying interference with bacterial growth.^111^ In Gram-positive and Gram-negative bacteria, sanguinarine inhibits cytokinesis by disrupting Z-ring assembly through binding to FtsZ and affecting the bundling of protofilamints.

Arborinine and graveoline exhibit inhibitory activity against *C. albicans* at 250 and 500 lg/ml, respectively. MIC/MFC indicated that these alkaloids played a bactericidal role. Analysis of gene and protein expression revealed effective effects for both compounds on ICL1 gene andprotein expression. Hence, arborinine and graveoline appear to be promising inhibitors of ICL1 in *C. albicans* glyoxylate cycles.^112^ A phytochemical investigation of the 90% ethanol aqueous extract of the bulbs of Crinum latifolium led to the isolation of three new crinane-type alkaloids, designated crinumlatines A-C (1–3). Compounds 1–3 exhibited some antimicrobial activity against the tested Gram-negative bacteria with minimum inhibitory concentration values less than 50 lg mL^−1^.^113^

Catharanthine was deduced first through in *silico* inhibition of efflux pump proteins MexA, MexB and OprM. Catharanthine-induced efflux pump inhibition may be useful for lowering antibiotic doses, reducing drug resistance, and increasing the efficacy of old antibiotics against multidrug-resistant bacteria.^114^

Plants selected for anti-mastitis protection contain higher concentrations of phytochemicals, with alkaloids of *A. sativum and B. persicum* showing significant antibacterial activity compared to that of the other selections. Additionally, ATCC strains show greater inhibition of phytochemicals of tested plant species compared to that of MDR bacterial strains.^115^

We can see that chanoclavine may make it easier to repurpose antibiotics that have become nonfunctional because of acquired bacterial resistance. By inhibiting efflux pumps, weakening the cell envelope, and increasing cell growth, the molecule synergizes with conventional antibiotics to target bacterial cell membranes or cell walls.^116^

### 3.3. Terpenoids

**Fig. 5:**
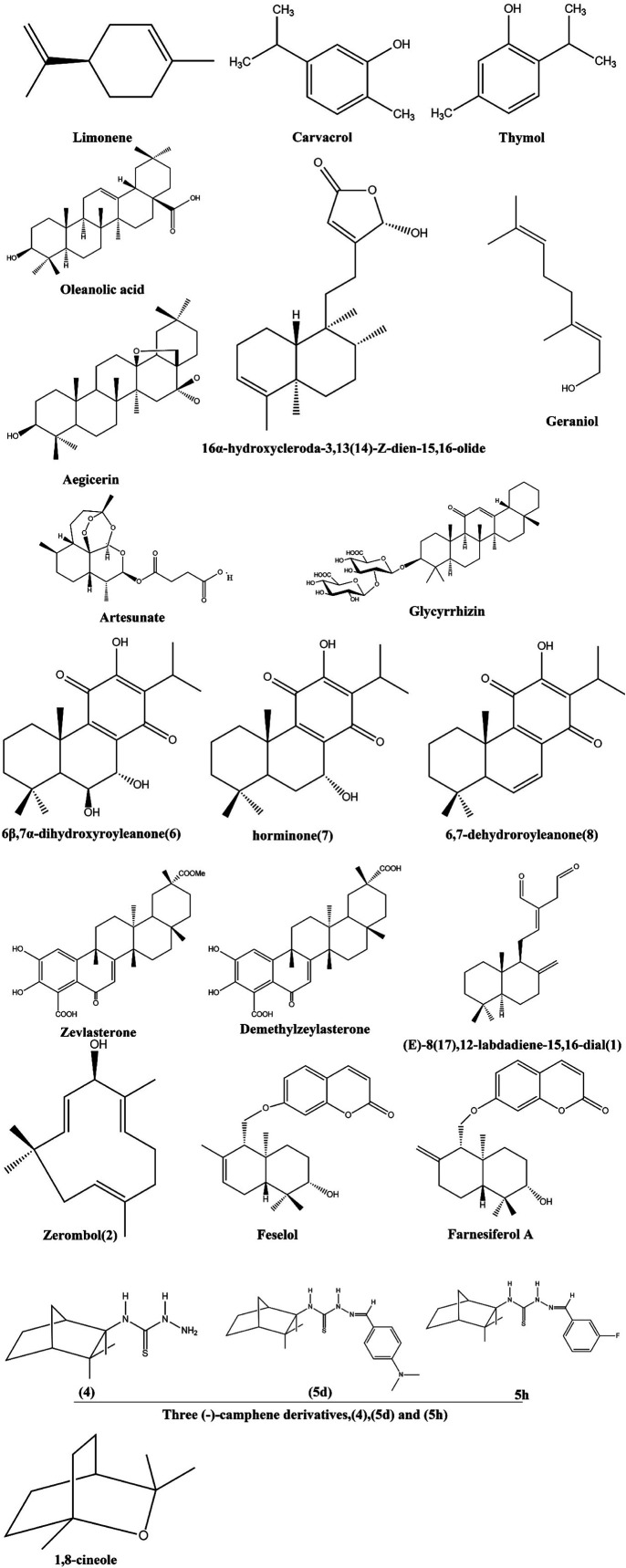
Structure of terpenoids having MDR modulatory activity.

Terpenoids, which are abundant in nature and produced in response to microbial attack, have huge potential as antimicrobial agents through a variety of mechanisms, such as disruption of membranes, anti-quorum sensing, inhibition of protein synthesis and ATP.^92,117,118^ Combinations of terpenoids and antimicrobials, such as combination therapy, have increased the effectiveness of treatment against multidrug resistant microorganisms by showing synergy (Fig. 5).

#### 3.3.1 Limonene

The antibacterial activity of limonene-containing essential oils has been demonstrated against resistant strains of *S. aureus*, *Salmonella enterica*, and *Listeria monocytogenes*. The mechanism may be due to the lipophilic properties of limonene. Limonene may cross the cell wall and alter the permeability of bacterial cell membranes.^119^ When *E. coli* cells were treated with limonene, the cell membrane was disrupted, cellular leakage occurred, and cell death occurred. Protein leakage, lipid leakage, and nucleic acid leakage confirmed that membrane damage and disruption of the permeability barrier of the cell occurred. In addition, the release of intracellular ATP suggested disruption of membrane barriers occurred. The interaction of limonene with DNA revealed its ability to unwind plasmids, which could eventually inhibit DNA transcription and translation.^120^ Various proteins and enzymes involved in transport, respiration, metabolism, chemotaxis, and protein synthesis were expressed differently, confirming the mechanistic role that limonene plays in these processes.

#### 3.3.2 Carvacrol and thymol

Both carvacrol and thymol are moderately effective against multiresistant *S. aureus* strains with the NorA, SA-1199 (wild type), and SA-1199B (overexpressed NorA) efflux pumps. Additionally, carvacrol and thymol potentiated the MIC of *norfoxacin* and *ethidium bromide*, increasing the effectiveness of these compounds against strains that carry the NorA efflux proteins SA-1199 and SA-1199B.^121^ Based on the docking results, the NorA binding pocket is involved in the interaction between carvacrol and thymol. It is confirmed that these terpenes compete with Norfoxacino and ethidium bromide for NorA binding sites.

#### 3.3.3 Oleanolic acid

Martins *et al.* (2011) isolated and tested six compounds, namely, uvaol, β-amyrin, oleanolic acid, catechin, epicatechin and monogalactosyldiacylglycerol. In studies involving a wide range of bacterial strains, oleanolic acid showed strong antimicrobial activity. In comparison to all of these compounds, uvaol reduced MRSA COL_OXA_'s resistance to the antibiotic oxacillin most effectively. Overexpressed efflux pump systems are responsible for the MDR phenotype of MRSA COL_OXA_. Uvaol inhibits EtBr accumulation and efflux by MRSA COL_OXA_ strains that are dependent on glucose levels, suggesting that the effect of uvaol on the efflux pump system of this organism appears to be the mechanism that contributes to its ability to reduce oxacillin resistance.^122^ Uvaol's effect on the efflux pump system of other MDR strains also contributes to reversing their resistance to given antibiotics.

#### 3.3.4 Thymol

Oil from *T. ammi* contains mostly thymol, which is among a class of natural phenolic monoterpenes known as biocides. Whether used alone or in conjunction with other biocides, such as carvacrol, thymol has a strong antimicrobial effect.^123^ Previous studies on Gram-positive test strains have found that compounded substances influence many physiological characteristics, including cytoplasmic membrane permeability, coagulase activity, and salt tolerance.

#### 3.3.5 16α-hydroxycleroda-3,13(14)-Z-dien-15,16-olide (CD)

CD from leaves of *Polyalthia longifolia* (Sonn.) were examined. By using Thwaites (Annonaceae) as RAM, current staphylococcal infection treatment strategies can be improved. CD significantly reduced the MIC of fluoroquinolones up to 16-fold (FICI 0.315-0.500). Combining CD with norfloxacin significantly (*p* < 0.01, *p* < 0.001) reduced the systemic microbial burden in blood, liver, kidney, lung, and spleen tissues of Swiss albino mice infected with *S. aureus*, norfloxacin alone or untreated control.^124^ Flow cytometry analysis clearly demonstrated that CD significantly inhibited EtBr efflux and extended its postantibiotic effects.

#### 3.3.6 Geraniol (Ger)

In aromatherapy, Ger is traditionally used to treat vaginal candidiasis since it is a monoterpene alcohol that comprises approximately 20% of geranium oil. Through transmission electron micrographs, we were able to observe depleted ergosterol levels and altered plasma membrane ATPase activity as signs of membrane tampering. The effect of Ger on cell walls has also been documented by spot assays with agents that perturb the cell wall and scanning electron micrographs.^125^ There seems to be an essential role for the calcineurin pathway in the antifungal effect of Ger because the calcineurin signaling mutant was highly resistant to Ger, whereas the strains overexpressing calcineurin remained resistant. Ger also causes mitochondrial dysfunction, iron homeostasis disturbances, and genotoxicity. Moreover, Ger inhibits both hyphal morphogenesis and biofilm formation, two of its leading virulence attributes.

#### 3.3.7 Aegicerin

Based on a colorimetric bioassay-guided fractionation protocol against *Mycobacterium tuberculosis* (MTB), the active constituent of an extract of the Peruvian plant *ClaVija procera* was identified as oleanane triterpenoid aegicerin^126^. Its MIC values ranged between 1.6 and 3.12 μg mL^−1^ against 37 different sensitive and resistant MTB strains (1H37Rv, 21 susceptible clinical isolates, 2 INH-resistant clinical isolates, and 13 MDR clinical isolates).

#### 3.3.8 Artesunate (ART)

According to recent studies, ART is effective at inhibiting efflux pump genes and may increase the effectiveness of various β-lactam antibotics in the treatment of MDR *E. coli*.^127^ Another study revealed that antimicrobial activity was not demonstrated by ART. However, a dramatic synergistic effect of ART and FQs was observed with (∑FIC) = 0.12–0.33. As a result of ART, MDR *E. coli* isolates accumulated DNR and expressed acrAB-tolC mRNA less than control isolates, but compared to the controls, qnrB and qnrS exhibited a greater expression.

#### 3.3.9 Glycyrrhizin (GLY)

GLY is a glycoconjugated triterpene derived from licorice root (Glycyrrhiza glabra), and the antimicrobial and anti-inflammatory effects of GLY can cause severe and difficult-to-treat keratitis *in vivo* in isolates of MDR organisms. Various mechanisms appear to be involved, including the MIC of ciprofloxacin being lowered, bacteria being permeabilized, swelling occurring, and clumps forming.^128^ Through the reduction of efflux pump activity and the increase in bacterial killing, ciprofloxacin has been shown to improve clinical outcomes.

#### 3.3.10 Ryleanones

The royleanones, which contain a *p*-benzoquinone C ring, *i.e.*, 6β,7a-dihydroxyroyleanone (6), horminone (7), and 6,7-dehydroroyleanone (8), showed mild activities against the MDR strain (MIC values 12.5 mg mL^−1^), and, distinctively, the MIC value of 7a-acetoxy-6b-hydroxyroyleanone (5) was 3.12 mg mL^−1^, which was the most potent antimycobacterial agent against the MDR strain and against the H37Rv strain with an MIC value of 25 mg ml^−1^.^129^ Based on these results, the presence of the 7a-AcO group in the B ring may be essential for increasing MDR-MTB activity.

#### 3.3.11 Conjugates

By computing docking scores for outlandish conjugates of SMZ and monoterpenes, effective agents against DHPs of five bacterial species can be identified. The docking scores for the monoterpenes or SMZ conjugates against five bacterial DHPs were also higher than those for monoterpenes or SMZ alone. In addition, conjugates have been shown to be safer antibacterial agents when both toxicity and LD50 values are taken into consideration. Conjugate 5 (SMZ + thymol) with docking scores greater than −10.0 kcal mol^−1^ was the most effective and safest antibacterial agent, while SMZ had docking scores of −8.46 and a high LD50 value of 3500 mg kg^−1^.^130^

#### 3.3.12 Farnesol

By inhibiting biofilm resistance, farnesol, a QSM secreted by *C. albicans*, affects the formation of biofilms. *C. albicans* has been shown to be inhibited not only on its ERG genes but also on its MDR1 gene, an important multidrug resistance gene.^131^ In addition to its many other effects on eukaryotic and prokaryotic cells, FAR also alters ABC efflux transporters, resulting in changes in resistance to azoles in *C. albicans* isolates.^132^ However, this effect is dependent on FAR concentrations.

#### 3.3.13 Zeylasterone and demethylzeylasterone (6-oxophenolic triterpenoids)

Zeylasterone and demethylzeylasterone (6-oxophenolic triterpenoids) obtained from the roots of Maytenus blepharodes showed potent activity against *Staphylococcus aureus*. Compared to demethylzeylasterone, zeylasterone showed superior activity against microbial cytoplasmic membranes, causing them to rupture. Ethyl acetate extracts from *Croton macrostachys* were tested for their antibacterial properties against *S. aureus* and antifungal properties against *Candida*.^133^ Five compounds were obtained, including two were lupane triterpenoids and three clerodane diterpenoids, with betulin and 12-oxo-hardwickic acid showing the greatest activity. It was found that lupeol isolated from the aerial parts of brillantaisia lamium and trichodesma amplexicaule inhibited bacterial and fungal growth significantly by causing membrane damage as a possible mechanism of action.

#### 3.3.14 Other terpenoids

Bioassay-directed isolation of secondary metabolites from the rhizomes of *Zingiber montanum* (*E*)-8(17),12-labdadiene-15,16-dial (1), zerombol (2) demonstrated antibacterial activity (MIC values 32–128 g mL^−1^) against a series of clinical isolates of MDR and MRSA.^134^

Feselol and farnesiferol A are sesquiterpene coumarins that are obtained by extracting aerial parts of *Ferula vesceritensis* (Apiaceae) in dichloromethane, which is associated with a model of the recombinant nucleotide binding site of *Cryptosporidium parvum's* enteropathogenic efflux pump.^135^

For the first time, the antibiofilm activity of 1,8-cineole against *E. coli* producing MDR ESBLs was demonstrated.^136^ There is no doubt that the compound is capable of causing substantial bacterial death in biofilm-attached as well as biofilm-released cells. The (−)-camphene derivatives tested in the present study, particularly (4) (5d) and (5 h), were shown to be promising anti-TB molecule scaffolds due to the low MIC values in acidic pH against the reference strain H37Rv and MDR *M. tuberculosis* clinical isolates, their low cytotoxicity and the synergism observed with PZA.^137^

All bacteria tested were inhibited by the highest polar fraction of Cistus ladaniferus essential oil, which contained mono- and sesquiterpene alcohols with MIC values ranging from 0.05 to 0.8 mg mL^−1^.^138^ There was high activity of this fraction against the MDR strsain *Enterobacter aerogenes* EA289, and its mechanism involves a distortion of the cell wall with detachment of the outer cytoplasmic membrane.

*In vitro* antibacterial activity of the essential oil of *E. camaldulensis* against multidrug resistant *A. baumannii* wound isolates, demonstrating the plant's traditional use for wounds. As constituents of the *E. camaldulensis* oil, polar terpene compounds and spathulenol may contribute to the observed antibacterial activity.^139^ Additionally, synergistic interactions of the antibiotics ciprofloxacin, gentamicin and polymyxin B with *E. camaldulsensis* essential oils were detected in this study for the first time against MDR *A. baumannii* isolates.

## 4. Conclusion and perspectives

In contemporary society, antibiotics have become an important part of everyday life; the wonders of antibiotics reveal how useful nature can be. Multidrug resistance (MDR) as well as cross-resistance to other drugs have become global concerns due to inappropriate use and overprescription of medications. In response to this emerging problem, we are entering an era of responsibility. Novel solutions are urgently needed to address this significant issue. Natural products are currently being investigated for their antimicrobial properties. Based on the current literature, it appears that there is an urgent need to coordinate efforts for meaningful research and for finding novel alternatives that utilize natural products.

The present study presents a wealth of information regarding the phytocomplexes and mode of action of purified antimicrobials that are isolated and purified from potential medicinal plants. Additionally, these components can be used to design new nutraceuticals or other drugs that are effective. New therapeutics will, however, be discovered by exploring the additional bioactivity of the corresponding species and genus.

## Conflicts of interest

There are no conflicts to declare.

## Supplementary Material
